# Cerebellar White Matter Abnormalities following Primary Blast Injury in US Military Personnel

**DOI:** 10.1371/journal.pone.0055823

**Published:** 2013-02-07

**Authors:** Christine Mac Donald, Ann Johnson, Dana Cooper, Thomas Malone, James Sorrell, Joshua Shimony, Matthew Parsons, Abraham Snyder, Marcus Raichle, Raymond Fang, Stephen Flaherty, Michael Russell, David L. Brody

**Affiliations:** 1 Department of Neurology, Washington University School of Medicine, St Louis, Missouri, United States of America; 2 Department of Radiology, Washington University School of Medicine, St Louis, Missouri, United States of America; 3 Department of Trauma Surgery, Landstuhl Regional Medical Center, Landstuhl, Germany; 4 Rehabilitation and Reintegration Division, US Army, San Antonio, Texas, United States of America; University of South Florida, United States of America

## Abstract

Little is known about the effects of blast exposure on the human brain in the absence of head impact. Clinical reports, experimental animal studies, and computational modeling of blast exposure have suggested effects on the cerebellum and brainstem. In US military personnel with isolated, primary blast-related ‘mild’ traumatic brain injury and no other known insult, we found diffusion tensor MRI abnormalities consistent with cerebellar white matter injury in 3 of 4 subjects. No abnormalities in other brain regions were detected. These findings add to the evidence supporting the hypothesis that primary blast exposure contributes to brain injury in the absence of head impact and that the cerebellum may be particularly vulnerable. However, the clinical effects of these abnormalities cannot be determined with certainty; none of the subjects had ataxia or other detected evidence of cerebellar dysfunction. The details of the blast events themselves cannot be disclosed at this time, thus additional animal and computational modeling will be required to dissect the mechanisms underlying primary blast-related traumatic brain injury. Furthermore, the effects of possible subconcussive impacts and other military-related exposures cannot be determined from the data presented. Thus many aspects of topic will require further investigation.

## Introduction

Blast-related traumatic brain injury (TBI) has been called the ‘signature injury’ of the wars in Iraq and Afghanistan. The effects of isolated primary blast injury on the human brain have been a source of tremendous debate and uncertainty since at least the time of World War I [1], [2]. Recently, two seminal studies have been published that described tau pathology and axonal injury in US military personnel with blast-related TBI [3], [4]. Most notably, Goldstein et al described a 34 year old military veteran (their case 2) with a history of two blast-related TBIs. These were reportedly isolated primary blast events, without impact (A. McKee, personal communication). Tau pathology consistent with chronic traumatic encephalopathy was described, but the extent of axon injury or other pathology was not noted in this case [4]. Surprisingly, we are unaware of any modern pathological reports describing the effects of primary blast-related TBI [1], [2]. The prior literature on the clinical and pathological effects of blast-related TBI have been reviewed extensively[5]–[9], but it is clear that numerous questions about these injuries remain unanswered. Most pathophysiological investigations of human blast-related TBI [10]–[18] including our own [10] have involved subjects with a combination of primary blast exposure and head impact, or involved subjects for whom the presence or absence of head impact was not reported. Simulation studies have suggested that there may be a specific vulnerability of the brain to blast exposure unrelated to other mechanisms of TBI [19], [20]. This vulnerability has been investigated in only a single case report [21], but not in a series of individuals with single, primary blast exposure and no previous history of TBI or other neurological disorders. These cases are quite rare but scientifically important to our understanding of blast-related TBI. In the current study we evaluated four such individuals in an attempt to better understand the specific contribution of the primary blast event.

Diffusion tensor imaging (DTI) is a newer imaging technique that involves the measurement of water diffusion in multiple directions [22]–[24]. In the white matter of the brain, water diffuses faster along the predominant fiber direction (axial diffusivity) and more slowly in perpendicular directions (radial diffusivity). This technique is especially useful for assessing the directional asymmetry of this diffusion (anisotropy) which is high in healthy axons and typically becomes reduced after injury [10], [25].

## Methods

All investigations were conducted according to the Declaration of Helsinki principles. All procedures were approved by the Washington University Human Studies Research Protection Office. Written informed consent was obtained from each participant prior to inclusion in the study.

US military personnel with primary blast-related traumatic brain injuries (**Table 1**) were recruited from the Rehabilitation and Reintegration Division of the US Army by advertisement (M. Russell). Through rigorous telephone-based screening of military personnel with previous primary blast exposure, we identified four individuals who had reportedly only sustained a single event consistent with traumatic brain injury.

**Table 1:** Characteristics of study participants.

Characteristic	Controls (N = 18)	Primary Blast TBI (N = 4)
**Age - yr**		
Median	31	30
Range	19–49	23–36
**Gender - no. (%)**		
Male	18 (100)	3 (75)
Female	0	1 (25)
**Branch of Service - no. (%)**		
Army	15 (83)	4 (100)
Air Force	2 (11)	0
Marine Corps	1 (0.05)	0
Navy	0	0
**Rank - no. (%)**		
Officer	2 (11)	0
Enlisted	16 (89)	4 (100)
**Theater of operation - no. (%)**		
Iraq	14 (78)	4 (100)
Afghanistan	4 (22)	0

The subjects had no past medical history of any illnesses that are known or could reasonably be expected to affect brain white matter. Specifically, no subject was known to have cerebrovascular disease, multiple sclerosis, hypoxic/ischemic brain injury, HIV, severe electrolyte disturbances, liver failure, renal failure, heart failure, alcohol abuse, or longstanding psychiatric condition. Furthermore, they had no other history of TBI, no prior events causing change in neurological status, and no life history of any event that could potentially have caused a concussion. Specifically they had not suffered falls, motor vehicle accidents, sports-related injuries, assaults, or other injuries to the head that resulted in notable changes in neurological function. Collateral histories were also obtained to corroborate information. However, the possibility that some had suffered sub-concussive injuries cannot be ruled out (please see discussion).

At the time of study evaluation, all primary blast TBI subjects were 2–4 years post exposure and had not been exposed to any additional blasts, had not endured subsequent TBI, were not abusing alcohol or drugs and had not previously done so. All injuries met Department of Defense clinical criteria for ‘mild’ TBI [26]. A control group was studied for comparison (n = 18). The control population consisted of returning military personnel from Iraq or Afghanistan who were clinically evaluated to be free of brain injury with no history of head injury, neurological or psychiatric disorder [10]. Control subjects were 6–12 months post deployment and denied abuse of drugs and/or alcohol.

Following our previously published methods, the evaluations consisted of neurological, neuropsychological, and psychiatric examinations followed by an MRI scan with DTI on a 1.5T Siemens Avanto scanner [10]. Imaging protocol included T1-weighted 1 mm isotropic, T2-weighted 1 mm isotropic, and T_2_*-weighted conventional images as well as a 2 averages of a 25 direction diffusion tensor imaging sequence at 2.5×2.5×2.5 mm resolution.

**Figure 1:**
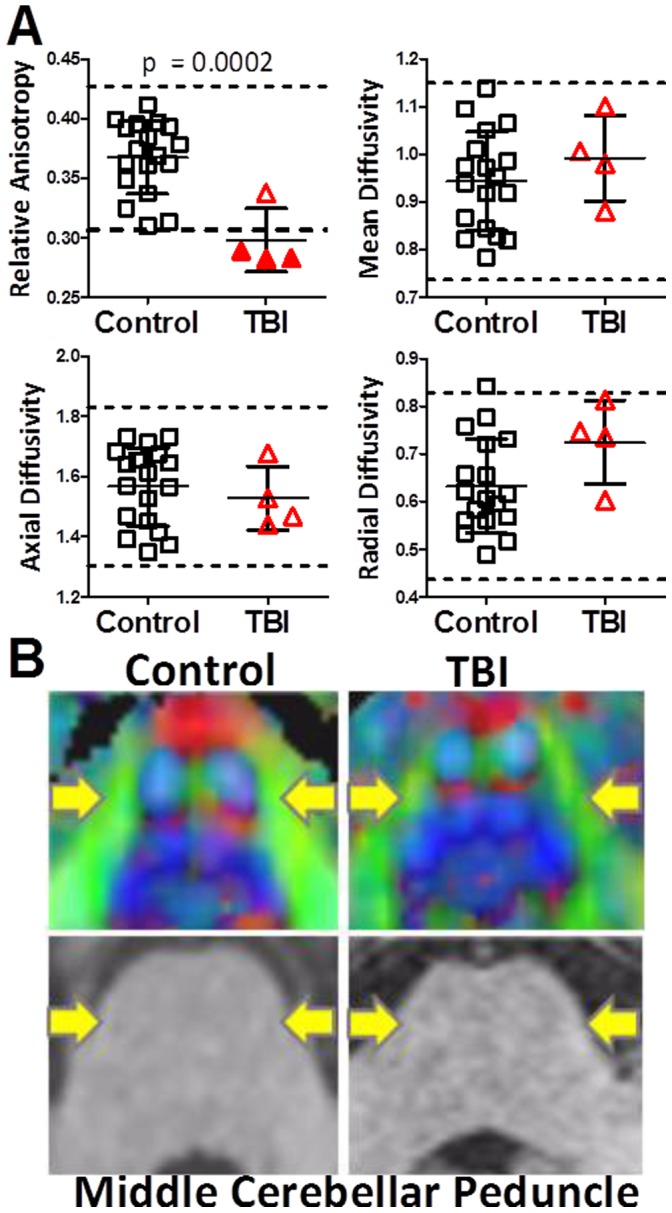
Diffusion tensor imaging (DTI) abnormalities in the middle cerebellar peduncle following a single primary blast exposure. A. DTI parameters in the bilateral middle cerebellar peduncles from controls and subjects with primary blast-related traumatic brain injury (TBI). Each symbol represents one subject. Dashed lines represent two standard deviations above and below the mean of the control group. Relative anisotropy is unitless. Axial, radial and mean diffusivities are plotted in units of 10^−3^ mm^2^/sec. B. Examples of DTI abnormalities (top panels) not apparent on conventional MRI (bottom panels). *Top Panels:* relative anisotropy maps. Yellow arrows indicate the middle cerebellar peduncles (shown in green on the DTI images). Intensity of color denotes relative anisotropy; brighter colors indicate higher relative anisotropy. Color denotes primary direction of diffusion; green: anterior-posterior, red: right-left, blue: rostral-caudal. *Bottom panels:* conventional T1 weighted MRI.

DTI data was initially analyzed following our previously published methods [10]. Briefly, after completion of post-processing and transformation into standardized space, regions of interest (ROI) were traced using a multislice approach through the entire 3-dimensional white matter region of interest. DTI parameters including relative anisotropy, axial diffusivity, radial diffusivity as well as the mean diffusivity were determined for each ROI. These regions included the genu, splenium, and body of the corpus callosum, the left and right cingulum bundle, left and right anterior limb of the internal capsule, left and right posterior limb of the internal capsule, left and right orbitofrontal white matter, left and right middle cerebellar peduncle and the left and right cerebral peduncles (please see Ref. 10, Figure 1).

Manually traced ROI DTI results were analyzed using Statistica software 6.0 (Statsoft). Relative anisotropy data was assessed using a two sided Hotelling’s T^2^-test followed by Bonferroni correction for multiple comparisons. For the 12 independent regions of interest, results were accepted as significant at p<0.0042 (i.e. 0.05/12). In planned post-hoc analyses, the other DTI parameters in the bilateral middle cerebellar peduncle (mean, axial, and radial diffusivity) were analyzed with two-sided Student’s t-tests. P-values reported have not been corrected for multiple comparisons. There was no evidence for deviations of the cerebellar region-of-interest data from normal distributions (Shapiro-Wilk W tests, p>0.05).

Subsequently, a whole brain coverage ROI analysis method based on DTIStudio [27] was employed in an exploratory fashion. For DTIStudio analyses, single subject images were aligned to a template atlas as previously published in a fully automated fashion [28]. For each subject, the DTIStudio analysis provided fractional anisotropy, radial diffusivity, axial diffusivity, mean diffusivity, and number of voxels for each of 130 regions of interest covering the entire brain. For the initial DTIStudio analyses, white matter was segmented using a fractional anisotropy (FA) threshold>0.20. DTI parameters were analyzed only in regions of interest analyzed where numbers of voxels did not differ between the TBI and control groups. This resulted in exclusion of the right and left medulla from further analyses. In a second round of analysis, regions of interest were segmented without a fractional anisotropy threshold. Statistically, DTIStudio data were assessed using 130 two-sided Student’s t-tests. A global test was attempted, but Hotelling’s T^2^-test did not converge. To appropriately correct for multiple comparisons over these 130 regions of interest, we used false discovery rate correction at p<0.05. This yielded essentially identical results to Bonferroni-Holm correction at p<0.1 (equivalent to one-sided tests at p = 0.05).

Subjects traveled to Washington University for in-person clinical assessments. The evaluations included a standardized neurological exam and structured interview designed for TBI patients (Neurobehavioral Rating Scale-Revised [29]), a neuropsychological test battery (see below), and psychiatric assessments including the Clinician-Administered PTSD Scale for DSM-IV (CAPS) [30] plus the Montgomery-Asberg Depression Rating Scale [31]. The standardized neurological exam and interview required approximately 1 hour per subject. The psychiatric assessments required approximately 2 hours per subject. Subjects took all usual medications. All tests were performed between 9 am and 5 pm in private, quiet, well-lighted rooms. All examiners were masked to other clinical information and imaging results. All examiners were clinicians who underwent standardized training in administering the assessments.

The neuropsychological test battery consisted of the Conners’ Continuous Performance Test II [32], a computer-based assessment of attention, impulsivity, reaction time, and vigilance; the Wisconsin Card Sorting Test [33], an assessment concept formation and mental flexibility; the Rey-Osterrieth Complex Figure Test [34]; a paper and pencil test of visual memory; the California Verbal Learning Test II [35], an assessment of verbal declarative memory; the 25 hole grooved pegboard test [36], an assessment of upper extremity motor speed and coordination; a timed 25 foot walk; the Trail Making test [37], an assessment of visual scanning, coordination and mental flexibility; the symbol digit modalities test [38], an assessment of working memory and processing speed; the controlled oral word association test [39], an assessment of verbal fluency; and the Wechsler Test of Adult Reading [40] as an estimate of pre-injury verbal intelligence. A relatively easy forced choice test embedded in the California Verbal Learning Test was used to assess adequacy of effort. Overall, the neuropsychological battery required approximately 2 hours per subject.

### Safety and Data Monitoring

A board certified psychiatrist (E. Nelson) was immediately available in case the CAPS examination exacerbated PTSD symptoms. No exacerbations requiring medical intervention occurred, though additional support from study staff was required on several occasions.

For clinical evaluations, the principal investigator audited 1 in 10 randomly selected subjects’ data sets to ensure that data was scored and entered correctly. These audits revealed only minor discrepancies in scoring criteria which were then corrected across the entire cohort of subjects.

## Results

At the time of the event causing isolated blast-related TBI, each subject endorsed a brief loss of consciousness, post-traumatic amnesia for 0–30 minutes, and/or change in neurological status (i.e. seeing stars, feeling dazed and confused). Sudden onset of headaches, nausea, sensitivity to noise and light was reported immediately following the event. The subjects reported that these acute post-concussive symptoms, with the exception of headache, largely resolved within 2 weeks to 1 month of the exposure. No injuries were sustained to the head, neck, torso, or extremities. The ISS score [41] for each subject was 0.

At the time of the evaluation 2–4 years after injury, primary blast subjects complained of headaches, irritability, anxiety, and problems with memory and attention. Neurological examinations were normal. Specifically, the subjects were found not to be ataxic or have extraocular movement abnormalities when examined by a board–certified neurologist (D. Brody). Likewise, neuropsychological testing performance was within normal limits in all subjects and controls (**Table 2**). All subjects performed well on a test of effort embedded in the California Verbal Learning Test. The psychometricians reported good apparent effort during testing. Evaluations for PTSD and depression identified one of the four subjects as meeting clinical criteria for both PTSD and depression. The clinician administered PTSD scale for DSM IV (CAPS) and the Montgomery-Asberg depression rating scale (MADRS) were used for these assessments.

**Table 2:** Neuropsychological test performance.

Test	Control (n = 18)	TBI (n = 4)	*P-value MWU test*
25-Foot Walk (seconds)* (Motor Strength, Balance, Coordination)*	5.2±2.1	4.7±0.4	0.79
Conners’ Continuous Performance Test II (T-scores)			
Omission Errors:* (Attention Lapses)*	54.5±21.2	49.7±14.2	0.47
Commission Errors:* (Impulsivity)*	50.9±10.5	56.9±15.9	0.33
Hit Rate:* (Reaction Time)*	49.4±11.2	48.9±14.5	0.77
Hit Rate Block Change:(*Sustained Vigilance)*	52.6±10.3	48.6±2.6	0.89
Wisconsin Card Sorting Test: Total Errors (T-scores)* (Concept Formation, Mental Flexibility)*	55.8±7.8	63.3±8.4	0.10
Rey-Osterrieth Complex Figure Test (T-scores)Delayed Recall *(Visual Memory)*	50.3±13.2	53.3±14.3	0.61
Wechsler Test of Adult Reading (standard scores)* (Estimate of Pre-injury Verbal Intelligence)*	97.6±12.6	103.5±6.6	0.55
California Verbal Learning Test II (standard scores)			
Long-Delay Free Recall* (Verbal Memory)*	0.0±0.9	−0.38±0.95	0.37
Total Intrusions* (Falsely Recalled Items)*	−0.44±1.5	0.25±0.87	0.47
List B vs. Trial 1 List A* (Proactive Memory Interference)*	0.11±1.1	−0.13±0.48	0.38
Grooved Pegboard (combined time & errors)* (Motor Speed & Coordination)*			
Dominant Hand	104±11.9	98.75±7.8	0.37
Non-Dominant Hand	109±14	96.5±5	0.11
Trail Making Test (seconds)			
Trails A time* (Visual Scanning, Coordination)*	24.8±5.6	26.7±4.2	0.37
Trails B time* (Trails A+Mental Flexibility)*	59.6±15.8	48.4±2.7	0.29
Symbol Digit Modalities Test (# correct written)* (Working Memory)*	54.4±9	51.3±2.9	0.22
Controlled Oral Word Association (# of words)Total Score: *(Verbal Fluency)*	32.2±7.2	36.8±7.6	0.37

DTI scans were abnormal in 3 of the 4 primary blast TBI subjects, based on quantitative comparisons with identically assessed US military controls (**Figure 1**). A global statistical comparison of relative anisotropy between primary blast TBI subjects and controls across 12 manually traced brain white matter regions of interest revealed a statistically significant difference between groups (p = 0.009, Hotelling’s T-test). This result was driven entirely by the findings in the middle cerebellar peduncle, as only this region differed between groups. A significant decrease in relative anisotropy (p = 0.0002, 1-sided student t-test) was observed in the primary blast TBI subjects (**Figure 1A**). This reduction in anisotropy is consistent with white matter injury. Notably, DTI revealed abnormalities in 3 of the 4 primary blast TBI subjects in comparison to controls in the middle cerebellar peduncle. We defined abnormalities as relative anisotropy more than 2 standard deviations below the mean of the controls. No other DTI parameter was found to be significantly different. The middle cerebellar peduncle abnormalities were apparent on close visual inspection of DTI scans, whereas conventional imaging in this region was normal (**Figure 1B**). A board certified neuroradiologist (M. Parsons or J. Shimony) closely evaluated the conventional (T1, T2, FLAIR, T2*) MR images and found no abnormalities in any of the primary blast TBI subjects or controls.

**Figure 2:**
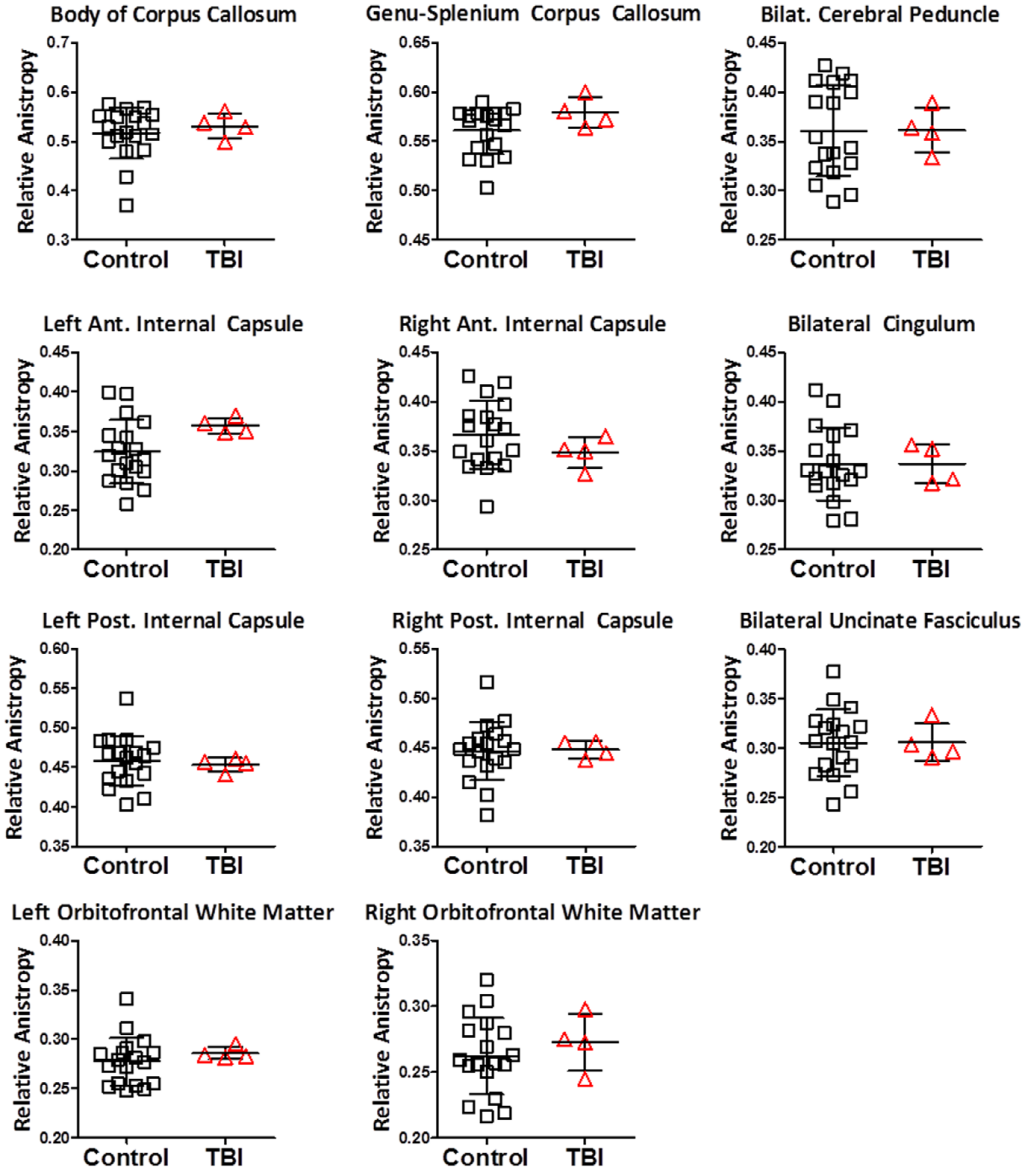
Diffusion tensor imaging was normal in 11 other brain regions in the 4 subjects with primary blast-related traumatic brain injury. Same subjects and controls as in Fig. 1.

A reduction in anisotropy was not found in any of the other 11 manually traced regions of interest in any of the primary blast TBI subjects (**Figure 2**). This provided initial evidence for a selective vulnerability of the cerebellar white matter to blast.

Surprisingly, we found that the size of the middle cerebellar peduncle appeared to be reduced in the primary blast TBI subjects. The volume of the middle cerebellar peduncle derived from the manually traced multi-slice region of interest covering the full 3-dimensional extent of this structure was 30,425±5,454 mm^3^ (mean, standard deviation) whereas it was 43,108±8,451 mm^3^ in the control group (p = 0.01, 2-sided t-test). No other regions differed in volume. The middle cerebellar peduncle reduction in volume could be due to injury-related white matter atrophy, as the subjects were assessed 2–4 years after blast exposure. However, in principle the volume reduction could also be due to bias in the manual region of interest tracing procedure despite our best efforts at prevention. Therefore, we turned to an additional unbiased approach.

**Figure 3:**
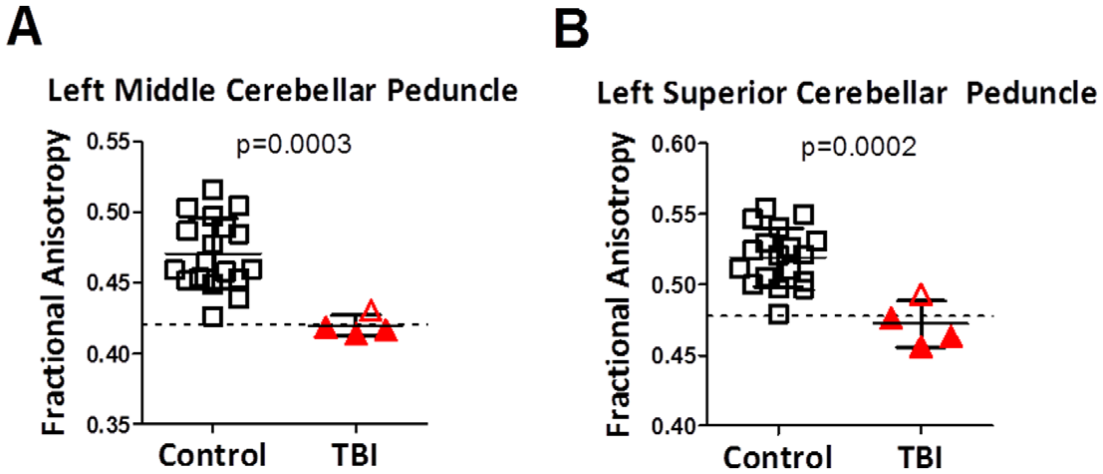
Diffusion tensor imaging abnormalities as assessed using DTIStudio in the same subjects. Note that fractional anisotropy is not numerically identical to relative anisotropy, though they fundamentally represent the same property of white matter. P-values represent uncorrected 1-sided student’s t-tests. Dashed lines represent two standard deviations below the mean of the control group.

A fully automated, unbiased analysis using DTIStudio revealed a similar reduction in fractional anisotropy in the left middle cerebellar peduncle in the primary blast TBI subjects compared to the controls (**Figure 3A**). Using a criterion for selecting white matter voxels as those with fractional anisotropy >0.2, there was no difference in the size of the left middle cerebellar peduncle in the primary blast TBI subjects compared with controls (p = 0.74, 2-sided t-test). This indicates that the difference between primary blast injury and control subjects is consistent across analysis methods and cannot be attributed solely bias in the tracing procedure, but also indicates that this region is not likely to be atrophic.

In an exploratory fashion, we asked whether other brain regions among the 130 covered by DTI studio differed between groups. After correction for multiple comparisons, only the left middle cerebellar peduncle (p = 0.0003, uncorrected 1-sided t-test, **Figure 3A**) and left superior cerebellar peduncle (p = 0.0002, uncorrected 1-sided t-test, **Figure 3B**) differed significantly between groups. There were also apparent differences in the left and right medulla but the data quality in these regions were not sufficient to interpret these results with confidence. Other regions with a trend towards reduced anisotropy in the primary blast injury subjects included the left parahippocampal gyrus (uncorrected p-value 0.002), right tapetum (uncorrected p-value 0.005), left globus pallidus (uncorrected p-value 0.011) and right globus pallidus (uncorrected p-value 0.012). These differences, however, were not significant after correction for multiple comparisons using either false discovery or Bonferroni-Holm methods. There were no regions in which anisotropy was found to be significantly higher in the primary blast injury subjects compared with controls, even at a trend level.

To ascertain whether the use of the FA>0.2 threshold in DTIStudio could have influenced these results, we next performed additional DTIStudio-based analysis with no FA threshold; all voxels were included. Interpretation of FA in gray matter regions or mixed gray and white matter regions is not straightforward, so we limited our analysis to the 68 brain white matter regions and excluded the 62 mixed gray and white matter regions. The unthresholded FA DTIStudio analysis largely confirmed our previous results: FA was reduced in the left middle cerebellar peduncle (p = 0.003, uncorrected 1-sided t-test) and left superior cerebellar peduncle (p = 0.0001, uncorrected 1-sided t-test). While the left middle cerebellar peduncle difference was no longer statistically significant after strict correction for multiple comparisons, the overall difference between groups (FA mean 0.406 in primary blast injury vs 0.452 in controls) was very similar to the difference found in the FA>0.2 threshold analysis (FA mean 0.418 in primary blast injury vs. 0.471 in controls). No additional regions were found to differ between groups using the unthresholded DTIStudio-based analysis methods. Again, there were no regions in which anisotropy was found to be significantly higher in the primary blast injury subjects compared with controls.

## Discussion

In summary, we found diffusion tensor imaging MRI abnormalities in the cerebellar white matter consistent with traumatic axonal injury in three of four US military personnel with clinical histories notable for isolated primary blast-related ‘mild’ traumatic brain injury. These results support the hypothesis that the cerebellum may be selectively vulnerable to primary blast effects. The cerebellum, brainstem, and orbitofrontal cortex were predicted to have the highest shear stresses during computational modeling of blast in comparison to other brain regions [19]. Previous studies and case reports have highlighted neuronal and metabolic changes in these same regions [3], [10], [21], [42]. Experimental animal models of blast [43] have also found axonal injury and neurodegeneration [4], [44]–[46] markers of inflammation [4], [44], [47] and hemorrhage [48] in the cerebellum and brainstem regions suggesting that there may in fact be a selective vulnerability in this area to blast exposure.

The clinical relevance of these cerebellar abnormalities remains to be determined. None of the subjects had ataxia, gait disturbance, objective evidence of cognitive impairment on a standard neuropsychological testing battery, or extraocular movement abnormalities apparent on bedside neurological examination. However, quantitative assessments of cerebellar-specific aspects of cognitive function, motor learning, vestibular function and extraocular movement were not performed because at the time that the study was designed and approved, we did not have specific hypotheses regarding cerebellar vulnerability to primary blast. Thus, more subtle vestibular disturbances and cognitive deficits in domains not tested that may be more sensitive to cerebellar lesions [49]–[51] cannot be ruled out. Clearly, future work will be required to definitively assess the clinical correlates of these cerebellar imaging abnormalities.

The diffusion tensor imaging acquisition approach used was selected to be consistent with our previous studies [10], allowing a direct comparison with an appropriate military control group. While higher resolution imaging at 3T with larger numbers of diffusion directions is currently available, the 1.5T, 25 direction methods we used were sufficient to allow adequate analysis of the major brain white matter tracts. The spatial and angular resolution of our approach are clearly limitations; studies involving higher resolution assessments of smaller white matter tracts and crossing fiber tracts will be an important area for future investigation.

A strength of this report is the use of two complementary analysis methods: manually traced region-of-interest analysis and fully automated region-of-interest analysis using DTIStudio. Manually traced region-of-interest analyses have the advantage that the anatomical landmarks and distinctions between gray and white matter voxels can be evaluated directly by analysts with extensive neuroanatomical knowledge and access to multimodal (T1, T2, FLAIR, DTI) images. They have the disadvantages of not providing full brain coverage, being time consuming, and being potentially biased: the group designations (injured vs. control) were known to the analysts. In contrast, DTIStudio-based analyses provide whole brain coverage, are unbiased, and require less analyst time, though they do require typically >12 hours of computer time on the DTIStudio servers for parcellation. DTIStudio-based analyses however have the disadvantage of not providing multimodal distinction between gray and white matter voxels. Therefore, FA thresholds are used to segregate white matter from gray matter, with the possibility of introducing bias in the process, e.g. injured white matter voxels with FA below the threshold are counted as gray matter and not analyzed. Thus, we view the general convergence of our results across these two methods as reassuring.

No objective documentation of blast intensities or distances from the blasts has been made publicly available for security reasons. As such, we cannot publicly comment on why the left cerebellar white matter tracts appeared more affected than the right cerebellar white matter tracts. We acknowledge that this limits the interpretation of the data presented. Another limitation is that these primary blast events could not be confirmed and screening relied on self report. A third limitation is that the effects of unreported subconcussive injuries [52], [53] or other military-related exposures cannot be determined with certainty. Nonetheless, these findings add to the evidence supporting the hypothesis that there may be a specific contribution of blast exposure to brain injury in the absence of head impact and that the cerebellum may be especially vulnerable.
